# Promises and pitfalls of safer smoking supply distribution in the United States: a thematic analysis of open-ended survey questions from harm reduction organizations

**DOI:** 10.1186/s12889-025-25599-4

**Published:** 2025-11-21

**Authors:** Kirstin Kielhold, William H. Eger, Angel K. Gomez, Tyler S. Bartholomew, Angela R. Bazzi

**Affiliations:** 1 Herbert Wertheim School of Public Health, University of California San Diego, 9500 Gilman Drive, Mail Code 0725, La Jolla, CA 92093 USA; 2 San Diego State University, San Diego, CA USA; 3 School of Medicine, University of California San Diego, La Jolla, San Diego, CA USA; 4 AIDS United, Washington, D.C USA; 5 University of Miami, Miami, FL USA; 6 Boston University School of Public Health, Boston, MA USA

**Keywords:** Analgesics, opioid, Heroin, Fentanyl, Overdose, Public health, Harm reduction, Community health services, Delivery of health care, Implementation

## Abstract

**Background:**

With rising prevalence of smoking unregulated drugs across the United States, there is a need for more community-based distribution of safer smoking supplies (e.g., glass pipes) to reduce health harms for people who smoke drugs. While smoking supplies are increasingly offered through harm reduction organizations (HROs), which have predominantly focused on addressing injection-related harms, little is known about HROs’ experiences implementing this relatively novel service.

**Methods:**

Between November 2023–January 2024, we administered a survey with closed- and open-ended questions about safer smoking supplies to representatives of U.S. HROs. Thematic analysis of responses to open-ended survey questions identified themes related to organizations’ motivations and experiences implementing safer smoking supplies, including challenges with addressing smoking drug use in the communities they serve.

**Results:**

Among 118 HROs responding to the survey, most were community-based organizations (74%) and had implemented safer smoking supplies (67%). From open-ended responses, the most common motivations for distributing safer smoking supplies included shifting local drug markets (e.g., from heroin to fentanyl) and increased community demand. Offering smoking supplies had positive implications for organizations, including improved engagement with previously underserved communities—such as people who primarily smoke drugs, and younger, unhoused, and racially and ethnically minoritized individuals. Funding restrictions presented a major challenge to implementing safer smoking supplies, and some respondents expressed concerns about potential negative health consequences of smoking drugs, including mouth and lung problems.

**Conclusions:**

Many U.S. HROs have implemented safer smoking supplies, resulting in improved engagement of previously underserved communities in their evidence-based prevention services. However, concerns remain regarding the feasibility, sustainability, and health implications of safer smoking supply distribution. In the context of rapid transitions from injecting to smoking unregulated drugs in communities across the U.S., support for HROs and research on shifting drug consumption patterns and impacts are urgently needed.

**Supplementary Information:**

The online version contains supplementary material available at 10.1186/s12889-025-25599-4.

## Introduction

The opioid and polysubstance use crises comprise one of the most prominent public health issues facing the United States (U.S.) and world today [1]. In the U.S., drug overdose is the leading cause of injury-related mortality [1], with the number of overdose deaths in 2021 being 23 times higher than it was 2013 [2]. While recent numbers have indicated some decline in overdose related deaths in recent months, these numbers are difficult to precisely estimate and are still unacceptably high [3, 4], with important disparities observed by race, ethnicity, and other socio-demographic factors [5, 6]. Prominent among the many factors contributing to the rise in overdose deaths is the increasing prevalence of intentional and unintentional consumption of fentanyl, a synthetic opioid many times more potent than heroin and morphine [7, 8]. In recent years, stimulant-related overdose deaths, particularly involving methamphetamine, have also surged, both independently and in combination with fentanyl [9, 10]. These overlapping trends have further exacerbated public health challenges related to the injection of unregulated drugs, including increased HIV and Hepatitis C virus (HCV) transmission and injection-associated wounds [11, 12].

In response to these evolving challenges, there has been a notable shift from injecting to smoking opioids and stimulants across the U.S. This shift was initially identified among people using fentanyl in San Francisco, CA, between 2018 and 2020, with individuals’ motivations to smoke instead of inject including difficulty finding veins, perceived reduced risk of overdose, and wanting to avoid health and social consequences of injecting (e.g., abscesses and stigma) [13]. A 2021 survey of people accessing opioid safe supply in Canada identified strong preferences for smoking opioids over injecting them, particularly among participants who had recently witnessed an overdose [14]. Following these initial reports, research has confirmed the rising prevalence of smoking unregulated opioids and methamphetamine across the U.S [15, 16].

Amidst these population-level shifts from injecting to smoking unregulated drugs, U.S. harm reduction organizations (HROs), predominantly syringe services programs (SSPs), have been increasingly distributing safter smoking supplies alongside other harm reduction supplies and services [17]. Safer smoking supplies typically encompass glass pipes (straight pipes for cocaine, bubble pipes for methamphetamine, and/or hammer pipes for opioids) or aluminum foil for smoking, rubber mouthpieces and lip balm to protect lips from burns, wooden push sticks and screens for handling drugs, and other sterile materials used to smoke drugs [17–19]. It is widely understood that the distribution of these supplies, predominantly sterile glass pipes, benefits the health and wellbeing of people who use drugs (PWUD) [18]. Indeed, a recent systematic review examining data on the utilization and delivery of safer smoking supplies globally through December 2022 found that safer smoking supply distribution was generally perceived as a form of harm reduction via reducing the sharing of smoking materials, clients’ injection frequency, HIV, HCV and overdose risk, and the health consequences of smoking drugs (e.g., burns, sores). Furthermore, a national study recently estimated that 44% of U.S. HROs offered safer smoking supplies in 2022, which was associated with having larger volumes of participant encounters and doses of naloxone distributed [20].

Despite the increasing distribution of safer smoking supplies in the U.S., the consequences of smoking drug use and safer smoking supply distribution in this context remains understudied. For example, in the systematic review cited above, only six studies examined the impact of safer smoking services on the individual health and well-being of PWUD; none of which were U.S. samples [18]. To our knowledge, only four studies exist on the distribution of safer smoking supplies in U.S.-based HROs [17, 19–21]. Of those, only two represent the experiences and perspectives of HRO staff distributing safer smoking supplies. Hence, beyond funding challenges [17, 19], little is known about what deters HROs to adopt safer smoking supplies, or related staff concerns about impacts on their organization and the clients they serve. To inform efforts to support HROs in adapting to population-level shifts in drug use in the context of the ongoing, severe opioid and polysubstance use crises, we sought to explore motivations for providing safer smoking supplies, perceived implications of doing so, and general challenges to addressing smoking drug use in a large sample of U.S. HROs.

## Methods

### Study design & sample

This study draws on responses to open-ended questions in an online survey administered to U.S. HROs from November 2023 to January 2024, as was previously described [17]. To identify and contact HRO representatives, we partnered with a national nonprofit organization, AIDS United, that is focused on improving the health of people with and at risk for HIV. We sent recruitment emails that briefly described the study and eligibility criteria (age 18 or older, English literacy), asked that only one representative per organization participate, and included a link to the electronic screening and informed consent process. After providing informed consent to participate, respondents were directed to a brief anonymous survey in Qualtrics (Provo, UT). Participants were not compensated. This research was carried out in compliance with the Helsinki Declaration and appropriate national guidelines. The Institutional Review Board at the University of California San Diego reviewed all study activities and provided a determination of Not Human Subjects Research.

### Data collection

As previously published [17], our survey included closed- and open-ended (i.e., short response) questions. Key survey domains included (1) organizational characteristics and contextual determinants (2), safer smoking supply implementation considerations and strategies (3), safer smoking supplies and services provided, and (4) sustainment and scale-up considerations. The final section of the survey included the following open-ended (i.e., short response) questions: (1) “To the best of your knowledge, why do you think demand for safer smoking supplies has increased?” (only asked of organizations reporting increased demand for safer smoking supplies); (2) “To the best of your knowledge, what do you consider to be some of the potential drawbacks of smoking drugs (compared to injecting drugs) for people who use drugs?” (asked of all organizations); and (3) “In your own words, please briefly describe the main benefits of offering safer smoking supplies at your organization” (only asked of those who reported offering safer smoking supplies; see Supplementary File). Respondents could decline to answer any question in the survey.

### Data analysis

We conducted an inductive analysis of responses to open-ended (i.e., short response) items following an “open coding” framework consistent with applied thematic analysis [22, 23]. Two team members, both graduate-level health services researchers with advanced training in qualitative methods and past qualitative research experience in the health sciences (KK and WE), independently reviewed all responses to the open-ended items to familiarize themselves with the range of content and generated a list of preliminary codes. Rather than using a predefined codebook, initial codes were developed through interpretative engagement with the data and attention to nuances of participant responses and experiences. The two team members then met to compare preliminary codes, reconcile discrepancies, and collaboratively develop a detailed codebook that included definitions and inclusion/exclusion criteria for each code. Both coders independently applied the codes to all responses in Microsoft Excel and met again to review and resolve any coding differences. One team member (KK) then conducted a final review of all coded data to ensure consistency and resolve any remaining discrepancies in consultation with the second coder (WE). Thematic analysis then involved three researchers (KK, WE, AB) reviewing relevant coded data (from codes on drug markets, harm reduction, awareness, regulations, health, access, sharing, overdose risk, expanding community, autonomy, and stigma) to identify overarching themes. Themes were developed through examining patterns and relationships among initial codes, allowing for the consolidation of related codes into broader, conceptually meaningful themes. Each team member independently drafted preliminary thematic summaries, which were then discussed as a group to develop consensus on the final themes to report. We considered thematic development complete when no new themes or refinements emerged during team discussions, suggesting consensus had been reached. Findings are described in detail and illustrated with representative quotes in the next section.

## Results

### Sample characteristics

Among the 118 U.S. HROs that completed the survey, represented regions included the West (*n* = 48; 41%), South (*n* = 28; 24%), Northeast (*n* = 21; 18%), and Midwest (*n* = 19; 16%; Table 1). The majority were non-profit community-based organizations (74%) and about a quarter were programs of city, county, or state health departments (26%). Most (72%) had been operating for over five years. The median number of unique clients served monthly was 300 (IQR: 148–647). The most commonly distributed supplies included naloxone (99%), syringes (92%), safer sex supplies (97%), basic first aid (92%), and referrals (90%).

**Table 1:** Characteristics of the sample of US harm reduction Organizations, November–December 2023, (*N* = 118)

Characteristic	Total (*N* = 118)
Region	
Midwest^a^	19 (16%)
Northeast^b^	21 (18%)
West^c^	48 (41%)
South^d^	28 (24%)
Missing	2 (1.7%)
Urbanicity*	
Urban	73 (62%)
Suburban	37 (31%)
Exurban/Semi-rural	30 (25%)
Rural	46 (39%)
Organization classification*	
Community-based organization/non-profit	87 (74%)
City, County, or State Health Department	31 (26%)
Other type of organization	6 (5%)
Funding*	
Federal funding	55 (47%)
State funding	98 (83%)
Foundation funding	53 (45%)
Fundraising funding	56 (48%)
Other funding	33 (28%)
Duration of operation	
Less than one year	2 (2%)
1–2 years	6 (5%)
3–5 years	24 (20%)
Greater than 5 years	85 (72%)
Missing	1 (0.8%)
Number of unique participants (per month) (Median, Interquartile Range)	300 (148, 647)
Offers safer smoking supplies	79 (67%)
Perceived benefits of smoking drugs (compared to injecting drugs)*	
Reduced risk of overdose	85 (72%)
Reduced risk of blood-borne infectious diseases (e.g., HIV, Hepatitis, bacterial infections)	107 (91%)
Reduced risk of skin infections or wounds	100 (85%)
Reduced stigma	76 (64%)
Missing	6 (5%)
Services Offered*	
Anal Administration Supplies	62 (53%)
Basic Clinical Services	42 (36%)
Basic First Aid	109 (92%)
Case Management/Housing Coordination Services	59 (50%)
Food	70 (59%)
Hepatitis C Testing	77 (65%)
Hepatitis C Treatment	27 (23%)
HIV Testing	84 (71%)
HIV Treatment	24 (20%)
Medication Assisted Treatment	67 (57%)
Mental Health Services	35 (30%)
Naloxone	117 (99%)
PrEP or PEP for HIV	41 (35%)
Referrals	106 (90%)
Safer Sex Supplies	114 (97%)
Safer Snorting Supplies	64 (54%)
STI Testing	58 (49%)
STI Treatment	38 (32%)
Syringes	109 (92%)

a. Includes: Illinois, Indiana, Iowa, Kansas, Michigan, Minnesota, Missouri, Nebraska, North Dakota, Ohio, South Dakota, Wisconsin

b. Includes: Connecticut, Maine, Massachusetts, New Hampshire, New Jersey, New York, Pennsylvania, Rhode Island, Vermont

c. Includes: Alaska, Arizona, California, Colorado, Hawaii, Idaho, Montana, Nevada, New Mexico, Oregon, Utah, Washington, Wyoming

d. Includes: Alabama, Arkansas, Delaware, Washington D.C., Florida, Georgia, Kentucky, Louisiana, Maryland, Mississippi, North Carolina, Oklahoma, South Carolina, Tennessee, Texas, Virginia, West Virginia, Puerto Rico

*‘Variable was ‘select all that apply’

***Acronyms used: *PrEP* Pre-exposure prophylaxis, *PEP* Post-exposure prophylaxis, *STI* Sexually transmitted infection

### Descriptive impacts of safer smoking supply distribution

Approximately two-thirds of HROs offered safer smoking supplies (*n* = 79; 67%), including alcohol swabs (91%), mouthpieces (85%), straight pipes (80%), screens/filters (80%), lip balm (82%), bubble pipes/“oil burners” (77%), educational materials on safer smoking practices (72%), foil (63%), and push sticks (57%) (Table 2). Many of these programs began distributing safer smoking supplies in the past 1–2 years (41%) and most reported increased enrollment of new clients since beginning to distribute safer smoking supplies (76%). Also, many reported decreased syringe distribution since starting to offer safer smoking supplies (29%). Among HROs reporting decreased syringe distribution since offering safer smoking supplies, 44% reported a 0–49% decrease, 17% reported a 50–99% decrease, and 39% were unsure or data was missing.

**Table 2:** Characteristics of and reported impacts of safer smoking supply distribution among organizations that provide safer smoking supplies (*n* = 79)

Characteristic	Total (*n* = 79)
Duration of providing smoking supplies	
Less than one year	14 (18%)
1–2 years	32 (41%)
3–5 years	19 (24%)
Greater than 5 years	9 (11%)
Missing	5 (6.3%)
Smoking supplies provided*	
Alcohol swabs	72 (91%)
Bubble pipes/oil burners	61 (77%)
Educational materials on safer smoking practices	57 (72%)
Foil	50 (63%)
Hammer pipes	36 (46%)
Lighters	14 (18%)
Lip balm	65 (82%)
Matches	5 (6%)
Mouthpieces	67 (85%)
Push sticks	45 (57%)
Safer smoking classes	7 (9%)
Screens/filters	63 (80%)
Straight pipes	63 (80%)
Missing	3 (4%)
Perceived change in enrollment of new participants since safer smoking supply implementation	
Increased	60 (76)
Decreased	0 (0.0)
Remained the same	8 (10)
Unsure or missing	11 (14)
Perceived change in syringe distribution since safer smoking supply implementation	
Increased	13 (17)
Decreased	23 (29)
Remained the same	25 (32)
Unsure or missing	18 (23)
Percent decrease in syringe distribution since offering safer smoking supplies^a^	
0–49%	10 (44)
50–99%	4 (17)
100%	0 (0.0)
Unsure or missing	9 (39)

*‘Variable was ‘select all that apply’

^a^ This question was only asked of participants that indicated there was a decrease in syringe distribution (*N* = 23)

### Qualitative findings

Our thematic analysis identified findings within the following three domains: (1) motivations for providing safer smoking supplies, (2) implications of providing safer smoking supplies, and (3) challenges addressing smoking drug use.

#### Motivations for providing safer smoking supplies

HROs described three primary motivations for offering safer smoking supplies including (a) changes in the unregulated drug markets and increased community demand, (b) safer smoking supplies as harm reduction, and (c) building trust and autonomy.

A *Changes in unregulated drug markets and community demand*: Participants frequently mentioned “changes” and “instability” in local unregulated drug markets as motivating the implementation of safer smoking supplies. Shifting drug markets (predominantly from heroin to fentanyl) led to HRO clients’ increased inhalation of unregulated opioids and stimulants, in turn increasing community need for safer smoking supplies. Examples of changes in the quality or content of local drug markets included seeing “*more fentanyl and xylazine in the supply*” (PID32; offers smoking supplies). Participants frequently described this increased variability and toxicity as precipitating a transition from injecting to smoking “*to mitigate… overdose risk*” (PID23; offers smoking supplies), based upon the belief “*that smoking will be safer*” (PID24; does not offer smoking supplies). Many participants also stated that “*more people are opting to smoke rather than inject now*” (PID1; offers smoking supplies) with statements such as, “*smoking has overall increased in our community*” (PID36; does not offer smoking supplies).
B *Safer smoking supplies as harm reduction*: Organizations’ desire to meet the harm reduction needs of their clients as they transitioned from injecting to smoking motivated the implementation of safer smoking supplies. Specifically, participants explained that “*supporting people transitioning from injecting to smoking*,* or stick[ing] with smoking*,* is a harm reduction intervention in itself”* (PID11; offers smoking supplies). Some participants discussed how clients’ transition from injecting to smoking is generally “safer” and that “*exploring harm reduction techniques for safer use*,* [like] intentionally shifting from using injection equipment*” (PID7; offers smoking supplies), could address clients’ overdose concerns or needs for better “*vein health*” (PID12; offers smoking supplies). While some participants discussed specific benefits of safer smoking supplies (e.g., decreased overdose and HIV/HCV transmission risk), most generally described smoking as “safer” than injecting rather than noting the specific health benefits of the supplies themselves.
C *Building trust and autonomy*: In addition to smoking being viewed as a “*safer way to consume [that] keeps people healthier and less at risk of death”* (PID103; offers smoking supplies), some participants emphasized how “*reliably offering clean pipes helps build trust with clients by demonstrating our commitment to harm reduction and the de-stigmatization of drug use*” (PID103; offers smoking supplies). Similarly, another participant explained:

*By offering [smoking] materials to support [people who use drugs] in making specific positive changes*,* we can ensure that folks are best equipped with knowledge and actual safer supplies. This can build trust and self-efficacy among clients and ultimately reduce fatality and injection related harms like HIV*,* HCV*,* sepsis*,* and bacterial infections”* (PID99; offers smoking supplies).

Additionally, a few participants mentioned the importance of offering safer smoking supplies for improving client autonomy, with one noting generally that “*it gives clients autonomy over how they choose to consume their substances*” (PID41; offers smoking supplies). Others more specifically explained how smoking could help clients avoid difficult relationships or people, as smoking requires less dependence on others; for example, smoking could be “*easier for women to navigate abusers trying to control them*” because it is “*an easier method to navigate*” (PID12; offers smoking supplies) than injecting.

#### Implications of providing safer smoking supplies

Participants from organizations offering safer smoking supplies described the implications of distributing them, including (a) increased engagement and new enrollments, and (b) expanding the reach of harm reduction services.

*A. Increased engagement and new enrollments*:The increased distribution of smoking supplies created increased community awareness of and engagement with HROs that offered them, with one participant commenting that, “*More folks have learned of our resources and are accessing our program solely for pipes*,*”* and “*people are more aware that these supplies exist and are excited to access them*” (PID88; offers smoking supplies). Another participant noted:

*Once we began giving them out [safer smoking supplies] and the word got out more and more people started coming for them. Most folks who did not inject didn’t know they could or didn’t feel comfortable coming to a needle exchange for supplies.* (PID31; offers smoking supplies)

Another participant mentioned, “We have had increase enrollment in our services and providing access, education, testing, support, etc. to more individuals” (PID58; offers smoking supplies).

*B. Expanding the reach of harm reduction services*:Expanded supply distribution led many HROs to reach communities that they previously did not serve due to a discrepancy between what they offered (e.g., syringes, medications for opioid use disorder) and what aligned with their communities’ needs. Participants explained that, with smoking supplies, they “*are meeting an entirely new set of people who can also benefit from the services we provide*” (PID3; offers smoking supplies). Other participants specifically noted the equity aspect of offering smoking supplies, as “*Black and Brown people access these [supplies] more*,* so it makes the program more equitable*” (PID8; offers smoking supplies). Similarly, one participant said, “*through distributing smoking supplies*,* we have expanded our reach…specifically to people who are younger*,* unhoused and unsheltered*,* Black and African-born*” (PID111; offers smoking supplies). The sentiments of being able to reach communities not traditionally reached by HROs was widely shared when asked of the benefits of offering safer smoking supplies. In addition to expanded reach in general, safer smoking supplies “*allows us to engage with more people and to offer more wrap around services”* (PID5; offers smoking supplies). One participant noted:

*We see people we have historically not been able to reach as a syringe exchange. We get to offer a whole other group of people services*,* testing*,* treatment*,* care*,* basic needs. Also reduces stigma for people who smoke drugs and recognizes that their health matters just as much as folks who inject* (PID31; offers smoking supplies).

Another participant described similar, specific benefits of reaching new communities with their services:

*More people in the community connect to our full-spectrum of healthcare services and referrals. More people in the community are carrying Naloxone due to stimulant users now knowing they can carry Naloxone too*,* which has led to more community [and] peer-based overdose reversals in the city* (PID14; offers smoking supplies).

#### Challenges addressing smoking drug use

Some participants discussed many positive implications of distributing safer smoking supplies without any drawbacks; however, others expressed specific concerns about potential (a) health concerns related to increased smoking drug use and (b) barriers to sustained implementation of safer smoking supplies, including client and system-level determinants.

A *Health concerns*: First, many participants questioned the safety of smoking drug use and whether safer smoking supply distribution would be good for individual or public health. Participants described specific health consequences of smoking unregulated drugs like issues related to the lungs, teeth, throat, mouth, and tongue. Other participants pointed out the overdose risks associated with smoking, noting an “*increased use of fentanyl*” (PID72; does not offer smoking supplies) that comes with smoking. Other participants brought up the false sense of security that could arise with transitioning from injecting to smoking, with one participant explaining, “*people think it is safer than it is*,* so they may be more reckless in their use*” (PID75; does not offer smoking supplies). Another participant said, “*just as many overdoses are being experienced by people smoking versus injecting*” (PID74; does not offer smoking supplies). Also, it was mentioned that there is a lack of education on how to smoke “safely” and concerns that “*people are more willing to share a pipe than a needle*” (PID23; offers smoking supplies), which has further negative health implications (e.g. COVID-19, HCV transmission). Finally, a participant from a health department-run organization explained, “*I am not certain giving away pipes to get people to use our services is good [for] public health. There is not strong evidence that giving away pipes reduces the spread of disease*,* [which is] the main reason why syringe services exist*” (PID53; does not offer smoking supplies).
B *Barriers to sustained implementation*: *(I) Client determinants*: The second most common concern among participants was related to stocking the necessary safer smoking supplies, and limited knowledge—among HRO staff and clients—of safer smoking practices. Multiple participants mentioned barriers like the “cost” and “sustainability” of attempting to provide safer smoking supplies as a major challenge. *(II) System-level determinants*: Another potential challenge mentioned was the stigma around smoking drug use, with multiple participants expressing concerns that smoking is not as easy to hide as injecting because it is more visible and draws “*unnecessary*” (PID101; offers smoking supplies) or “*unwanted attention to the person*” (PID118; does not offer smoking supplies). Another participant mentioned, “*it is less discrete than injecting*,* creating more push back from community members*” (PID108; offers smoking supplies). Moreover, others believed that people who smoke drugs have fewer legal protections than people who inject drugs, as they are not covered by the same syringe paraphernalia laws, as one participant noted, “*sadly*,* smokers are not protected by the syringe exchange law*” (PID66; offers smoking supplies).

## Discussion

Harm reduction organizations (HROs) play an essential role in improving individual and community level health and safety for people who use drugs (PWUD). Historically, HROs’ ability to create safe, non-stigmatizing spaces for the distribution of essential, lifesaving prevention supplies to those in need has proven effective for HIV prevention [24]. Expanding beyond the historic focus on HIV prevention, HROs have been effective in addressing many additional health consequences of unregulated drug use, including HCV prevention and treatment [25], linkage to drug treatment [26], reducing emergency department use [27], and naloxone distribution [28]. Organizations’ more recent expansion into safer smoking supply distribution represents an evolution in contemporary harm reduction [17], yet significant structural and policy challenges are apparent. We found that, motivated by changing drug markets and community demand, HRO representatives perceived many positive implications of offering safer smoking supplies, including increased engagement of new clients and traditionally underserved communities. Despite these positive impacts, barriers such as funding restrictions and stigma challenge the provision of safer smoking supplies, and some program representatives expressed concerns about the health consequences of smoking drugs, such as respiratory issues.

We found that organizations had many motivations for offering safer smoking supplies such as shifting drug markets (e.g., increased fentanyl contamination) and community demand necessitating new harm reduction responses. A 2023 review of safer smoking practices found that many individuals perceived smoking (compared to injecting) to be helpful for reducing the risks of overdose and HIV and HCV acquisition [18]. While some evidence on the overdose risk reduction role of smoking is emerging [29], there is clear community demand for safer smoking supplies, and syringe services programs and other HROs are responding. SSPs’ harm reduction model of “meeting people where they are at” has made them essential for responding to community need; for example, with the overdose crisis worsening during the COVID-19 pandemic, organizations increased naloxone distribution, adapted overdose prevention education, and increased their distribution of fentanyl test strips [28]. Our findings highlight HROs’ ability to respond to evolving community needs and demand by intentionally offering safer smoking supplies.

We also found that many organizations’ representatives perceived a relationship between offering safer smoking supplies and expanded program reach, including increasing enrollments of new participants and engagement of diverse, historically underserved communities (e.g. racial and ethnic minorities, unhoused individuals, younger individuals) who continue to face rising overdose-related mortality. Studies have found that people who inject are less likely to be Black [30] and higher percentages of Black individuals only smoke compared to White individuals [31], suggesting that criminalization of safer smoking supplies disproportionately impacts Black communities. In light of evidence highlighting the racial disparities in overdose trends [5, 32], it is extremely important to reach these communities. Our study found that nearly 100% of HROs distributed naloxone, suggesting that increasing engagement with these settings may have an important potential impact for racial justice and to reduce overdose disparities. Expanding HRO reach is critical for public health, as they connect participants with numerous evidence-based prevention supplies (e.g., syringes, naloxone) and services, which will be critical for specifically addressing rising overdose deaths among these underserved communities. Our findings build on the literature suggesting that further integration of safer smoking supply distribution within HROs can reach more diverse and marginalized community members while giving them access to other lifesaving services provided in these low-barrier settings [19, 20].

Despite the benefits described by many participants, we also identified some mixed perspectives and concerns regarding known and suspected health risks of smoking. Concerns about potential health harms from smoking unregulated drugs included oral, mouth, and lung health implications, which HROs are not traditionally prepared to address. Others questioned the limited evidence on smoking as an overdose prevention strategy. While some preliminary evidence suggests that people who solely smoke fentanyl experience fewer non-fatal overdoses and skin and soft tissue infections [29], potentially leading to lower overall mortality [33], limited longitudinal evidence exists on the comparative risks of smoking versus injecting unregulated drugs, highlighting a substantial research gap. The rise in smoking of methamphetamines and opioids has contributed to a 74% increase in the percentage of overdose deaths with evidence of smoking nationally between 2020 and 2022 [34]. Some evidence suggests that people who inject heroin, methamphetamines, and cocaine have higher rates of dependence and co-occurring physical and psychological problems compared to other routes of administration like smoking [35]. Longitudinal research on short, medium, and longer-term health outcomes of smoking drug use, and more in-depth qualitative research with people who smoke drugs and their perspectives and experiences (e.g. potential social harms), is needed.

From the short-answer responses analyzed here, we also confirmed the importance of funding and legal barriers to safer smoking supply distribution that were identified in our previous analysis of close-ended (i.e., quantitative) data from this survey of HROs, in which over half (58%) of organizations endorsed “insufficient funds” as a barrier to safer smoking supply implementation [17]. Additionally, while some U.S. state laws legalize HROs and the supplies they distribute, there are still many barriers to accessing and utilizing harm reduction services for PWUD. For example, in some U.S. states, possession of syringes is criminalized by drug paraphernalia laws [36]. This is also true for some safer smoking supplies, such as glass pipes, which are criminalized in even more states than syringes [37]. As one HRO representative noted in our study, people who smoke drugs may not be offered the same protections under the paraphernalia statues as people who inject, underscoring the need to reform drug paraphernalia laws to explicitly allow for the distribution of safer smoking supplies.

Our study has several major limitations worth noting. First, we surveyed a convenience sample of programs that may not generalize to all U.S. HROs; we received 118 responses, which is but a fraction (~ 20%) of known U.S. SSPs [38]. However, we had diverse organizational and geographic representation that supports the transferability of our findings. Second, for this analysis, we relied on short-answer responses to open-ended survey questions, which, unlike traditional qualitative methods, are subject to many important limitations (e.g., the topics of the questions were specific, and we had no ability to ask follow-up or probing questions following brief or unclear responses or interesting or insightful remarks). Our applied thematic analysis focused on systematically summarizing key topics across participants’ responses rather than generating deeply interpretive patterns of shared meaning; this orientation shapes both the nature of our findings and the quality criteria appropriate for evaluating them. Relatedly, as with all self-reported data, there is a potential for social desirability bias, and unlike traditional qualitative interviews, we had no opportunity to build rapport or trust with participants in the context of this survey. Overall, while this study provides some insight into organizations’ perspectives on safer smoking supply distribution, we did not quantify the prevalence of different perspectives, as our use of open-ended questions was aimed at exploring the breadth of perspectives rather than their frequency. Additional research, including studies with larger and more representative samples, should evaluate the prevalence of HROs’ safer smoking supply distribution, investigate impacts on health outcomes, contribute to the development of distribution guidelines, and test dissemination strategies to translate evidence to decision-makers who may be unaware, uncomfortable, or even opposed to harm reduction. Finally, research assessing cost-effectiveness and other implementation determinants and impacts of expanding safer smoking supply programs is needed to inform policy reform that strengthens the legal and funding environment to support implementation and sustainability.

Ultimately, in addition to describing organizational motivations and perceived impacts of implementing safer smoking supplies, our study offers several unique contributions to the literature. Most prior research on safer smoking supply distribution has been limited to smaller pilot studies without comparison groups or focused primarily on quantitative metrics [17, 19, 20]. In contrast, we present qualitative findings from a large, geographically diverse sample of U.S. HROs, offering a broader view of real-world implementation. Furthermore, we extend existing literature by identifying both positive implications, such as expanded engagement with historically underserved communities, and potential concerns, including uncertainty around the health impacts of increased smoking drug use and the logistical and legal challenges of sustained implementation. These findings offer critical insights for public health efforts aiming to support HROs in navigating shifting drug use trends while advancing equity and client safety.

## Conclusion

In this qualitative analysis of open-ended survey questions, we found that many U.S. HROs were motived to offer safer smoking supplies by shifting drug markets and community demand, and representatives perceived positive implications for their programs including expanded organization reach into larger and historically underserved communities. However, policy and funding restrictions present challenges to offering such supplies, and some program representatives expressed concerns about the health consequences of smoking unregulated drugs that remain unaddressed. Our findings highlight the need for public health leaders, policymakers, and funders to prioritize research and programs (including those with built-in evaluation mechanisms) focused on safer smoking supply distribution. HROs are effective and necessary in mitigating the harmful impacts of the evolving drug crisis. Given the alarmingly high overdose rates and the continuing drug market evolution, safer smoking supply distribution should be seen as an urgent and critical priority.

## Supplementary Information

Supplementary Material 1.

## Data Availability

The dataset used and/or analyzed during the current study is available from the corresponding author, William H. Eger (weger0735@sdsu.edu), upon reasonable request.

## Abbreviations

HROs: Harm reduction organizations
HCV: Hepatitis C virus
PWUD: People who use drugs
SSPs: Syringe services programs
U.S.: United States
