# Supplementary material for: Promises and pitfalls of safer smoking supply distribution in the United States: a thematic analysis of open-ended survey questions from harm reduction organizations
Source: BMC Public Health. 2025 Nov 21;26:360. doi: 10.1186/s12889-025-25599-4 (PMC12849471; doi:10.1186/s12889-025-25599-4)
Supplement: Supplementary file 1 — Supplementary Material 1. [file 12889_2025_25599_MOESM1_ESM.docx]

Safer Smoking Supply Implementation at SSPs – Open-Ended Survey Questions

Q1. To the best of your knowledge, why do you think demand for safer smoking supplies has increased? *Please briefly describe in your own words below.*

________________________________________________________________

________________________________________________________________

________________________________________________________________

________________________________________________________________

Q2. To the best of your knowledge, what do you consider to be some of the potential drawbacks of smoking drugs (compared to injecting drugs) for people who use drugs? *Please briefly describe below.*

________________________________________________________________

________________________________________________________________

________________________________________________________________

________________________________________________________________

Q3. In your own words, please briefly describe the main benefits of offering safer smoking supplies at your organization?

________________________________________________________________

________________________________________________________________

________________________________________________________________

________________________________________________________________
